# Unveiling Bladder Cancer Prognostic Insights by Integrating Patient-Matched Sample and CpG Methylation Analysis

**DOI:** 10.3390/medicina60071175

**Published:** 2024-07-19

**Authors:** Chanbyeol Kim, Sangwon Oh, Hamin Im, Jungsoo Gim

**Affiliations:** 1 Department of Biomedical Science, Chosun University, Gwangju 61452, Republic of Korea; cbvely01@gmail.com (C.K.); dpsej1031@chosun.kr (S.O.); 20210628@chosun.kr (H.I.); 2 AI Convergence College, Chosun University, Gwangju 61452, Republic of Korea; 3 BK FOUR Department of Integrative Biological Science, Chosun University, Gwangju 61452, Republic of Korea; 4 Well-Ageing Medicare Institute, Chosun University, Gwangju 61452, Republic of Korea; 5 Asian Dementia Research Initiative, Chosun University, Gwangju 61452, Republic of Korea

**Keywords:** bladder cancer, DNA methylation, prognostic biomarkers, survival analysis, personalized medicine

## Abstract

Bladder cancer prognosis remains a pressing clinical challenge, necessitating the identification of novel biomarkers for precise survival prediction and improved quality of life outcomes. This study proposes a comprehensive strategy to uncover key prognostic biomarkers in bladder cancer using DNA methylation analysis and extreme survival pattern observations in matched pairs of cancer and adjacent normal cells. Unlike traditional approaches that overlook cancer heterogeneity by analyzing entire samples, our methodology leverages patient-matched samples to account for this variability. Specifically, DNA methylation profiles from adjacent normal bladder tissue and bladder cancer tissue collected from the same individuals were analyzed to pinpoint critical methylation changes specific to cancer cells while mitigating confounding effects from individual genetic differences. Utilizing differential threshold settings for methylation levels within cancer-associated pathways enabled the identification of biomarkers that significantly impact patient survival. Our analysis identified distinct survival patterns associated with specific CpG sites, underscoring these sites’ pivotal roles in bladder cancer outcomes. By hypothesizing and testing the influence of methylation levels on survival, we pinpointed CpG biomarkers that profoundly affect the prognosis. Notably, CpG markers, such as cg16269144 (PRKCZ), cg16624272 (PTK2), cg11304234, and cg26534425 (IL18), exhibited critical methylation thresholds that correlate with patient mortality. This study emphasizes the importance of tailored approaches to enhancing prognostic accuracy and refining therapeutic strategies for bladder cancer patients. The identified biomarkers pave the way for personalized prognostication and targeted interventions, promising advancements in bladder cancer management and patient care.

## 1. Introduction

Bladder cancer (BLCA), despite its relatively low global incidence ranking (the 10th most commonly diagnosed cancer worldwide) and relatively high 5-year survival rate [1] compared to other malignancies, presents a significant healthcare challenge globally. Although BLCA shows a relatively high 5-year survival rate when diagnosed and treated in its early stages, the disease’s incidence is steadily rising, and advanced stages are associated with significantly higher mortality rates due to late diagnosis and the aggressive nature of the disease [2]. This dual nature underscores the critical need for enhanced diagnostic strategies and effective treatments to mitigate the impact of advanced BLCA stages on patient outcomes. Bladder cancer can be categorized into two main types: muscle-invasive bladder cancer (MIBC) and non-muscle-invasive bladder cancer (NMIBC). MIBC is characterized by the spread of cancer into the detrusor muscle of the bladder wall, which is indicative of a more aggressive disease course [3]. In contrast, NMIBC remains confined to the inner layers of the bladder wall and generally has a better prognosis [3,4]. Despite these distinctions, both forms of bladder cancer require diligent monitoring and treatment due to their potential to progress and recur. Effective management of NMIBC often involves intravesical therapy to reduce recurrence rates, while MIBC frequently necessitates more aggressive treatments, including radical cystectomy and systemic chemotherapy [3,4]. However, despite advances in treatment modalities, including surgery, immunotherapy, chemotherapy, and radiotherapy, the overall prognosis for BLCA patients remains unsatisfactory, particularly due to the heterogeneity of the disease and its delayed diagnosis in many cases [5,6,7]. In cases where diagnosis is delayed, despite aggressive treatment modalities such as surgery, immunotherapy, chemotherapy, and radiotherapy, the 5-year overall survival (OS) rate for BLCA, including both non-muscle-invasive and muscle-invasive forms, remains unsatisfactory, with a median OS of approximately 14 months [4]. Consequently, swift diagnosis and a robust prognosis that can accurately predict disease progression and guide personalized treatment strategies are essential.

Recent research has increasingly focused on epigenetic alterations, particularly DNA methylation, as potential biomarkers for various cancers, including BLCA [8]. Bladder cancer exhibits distinct DNA methylation patterns, notably including both hypo- and hypermethylation events. Promoter hypermethylation is particularly prevalent [9], often leading to the silencing of tumor-suppressor genes and contributing to cancer development and progression. Several studies have identified specific genes exhibiting these methylation patterns in bladder cancer. Research has highlighted genes such as RARβ and DAPK that frequently undergo promoter hypermethylation in bladder cancer [10]. Conversely, genes such as c-MYC have been identified as exhibiting hypomethylation patterns, potentially leading to their overexpression and oncogenic activity in bladder cancer [11]. DNA methylation, primarily occurring on cytosines, is less susceptible to storage conditions compared to proteins or RNA, ensuring biomarker stability [12]. Aberrant cytosine methylation can trigger mutations in genes driving tumor initiation [13], with previous studies demonstrating the impact of CpG methylation inhibition on tumorigenesis in BLCA [14]. Hence, understanding the aberrant CpG methylation patterns associated with bladder cancer progression holds promise for identifying novel prognostic markers and therapeutic targets [8].

However, the complexity of BLCA heterogeneity poses a significant challenge in biomarker discovery. Bladder tumors, like most other tumors, exhibit substantial intratumoral and intertumoral heterogeneity, with diverse molecular profiles and clinical behaviors observed even within tumors of the same histological subtype [15]. A recent study explored single-cell–level analysis, although its practical application in clinical settings remains nascent [16]. To address this challenge, matched sample analysis, involving the comparison of tumor tissue with adjacent normal tissue from the same patient, offers a valuable approach to minimize confounding factors and identify cancer-specific alterations.

In this study, we aim to explore the role of CpG methylation dynamics as prognostic biomarkers in BLCA, considering the context of cancer heterogeneity and the importance of matched sample analysis. By minimizing individual differences among patients, we deem the significance of our findings robust despite a small sample size. Beginning with the identification of differentially methylated CpG markers between paired samples of normal and tumor tissue, we employed a stepwise feature-selection procedure to isolate the most promising prognostic marker for BLCA.

## 2. Materials and Methods

### 2.1. Study Sample

This study utilized the sample from The Cancer Genome Atlas (TCGA) project, specifically the TCGA-BLCA dataset from the National Cancer Institute GDC Data Portal of the National Institutes of Health (NIH) in the United States as of 2022. To minimize cancer heterogeneity and focus on extreme survival outcomes, specifically death, matched pairs of cancer cells and adjacent normal cells from 10 patients were selected as the Discovery Phase dataset based on the sample type variable, which categorized the samples as primary tumors and normal solid tissue. Independent data from 275 bladder cancer patients, excluding these 10 patients, were utilized as the Survival Validation Phase dataset (Table 1). Notably, at the time of data acquisition in 2022, only 10 matched extreme cases were available for download, which influenced the initial patient selection for this study.

### 2.2. Methylation Data

DNA methylation data were available for the TCGA-BLCA sample, and the preprocessed individual-level data files were downloaded as .txt files from the TCGA portal. We initially downloaded data from 10 cancer patients along with their corresponding normal matching tissues. The individual datasets were combined to create a dataframe and structured to pair cancerous and normal tissues for each CpG marker from these 10 patients, and this was utilized for candidate marker selection in the Discovery Phase. Subsequently, methylation data from 275 independent cancer patients were downloaded and preprocessed into a general data structure containing CpG markers and sample IDs. This dataset was used for validation and survival analysis. From these datasets, we employed the following three-step biomarker selection analysis.

### 2.3. Primary Step: Candidate Biomarker Selection

In the first step of biomarker selection, paired sample tests were conducted to compare the methylation levels between normal cells and cancer cells using data from 10 deceased bladder cancer patients across approximately 480,000 CpG markers. The significance level was adjusted to 1% using the Bonferroni–Hochberg method for multiple comparisons to select candidate biomarkers. Specifically, markers exhibiting significant methylation differences between normal and cancer cells, with substantial effect sizes, were chosen as primary candidates, ensuring that there were no overlapping methylation levels across all 10 patients.

### 2.4. Secondary Step: Survival Validation

A secondary selection process was carried out for 6689 initially selected biomarkers to validate their association with survival using independent bladder cancer patient data. This phase included two analyses: Cox regression analysis for each marker to identify its association with survival time and gene set analysis, using the selected biomarker set to finalize the candidates. Cox regression analysis utilized a dataframe containing survival status, survival time (for deceased patients), and follow-up duration (for surviving patients) for 275 bladder cancer patients. Each marker was filtered based on a significance level of 1%. Gene set analysis focused on mapping CpG markers to genes using metadata, particularly targeting the GOTERM_BP_DIRECT pathway and emphasizing biologically relevant functions with significance levels set below 0.05.

### 2.5. Third Step: Threshold Determination

In the final step, the optimal prognostic marker was determined using the three final candidate genes mapped to their associated CpG markers. Initially, CpG markers linked with their final candidate genes were identified. Kaplan–Meier analysis followed the binary classification (hypomethylation/hypermethylation) based on each marker’s methylation status. The threshold for binary classification was iteratively adjusted to find the lowest significance level while avoiding extreme sample sizes in each classification group, thus serving as the final threshold.

## 3. Results

### 3.1. A Three-Step Strategy for Prognostic Biomarker Discovery in BLCA

**Figure 1:**
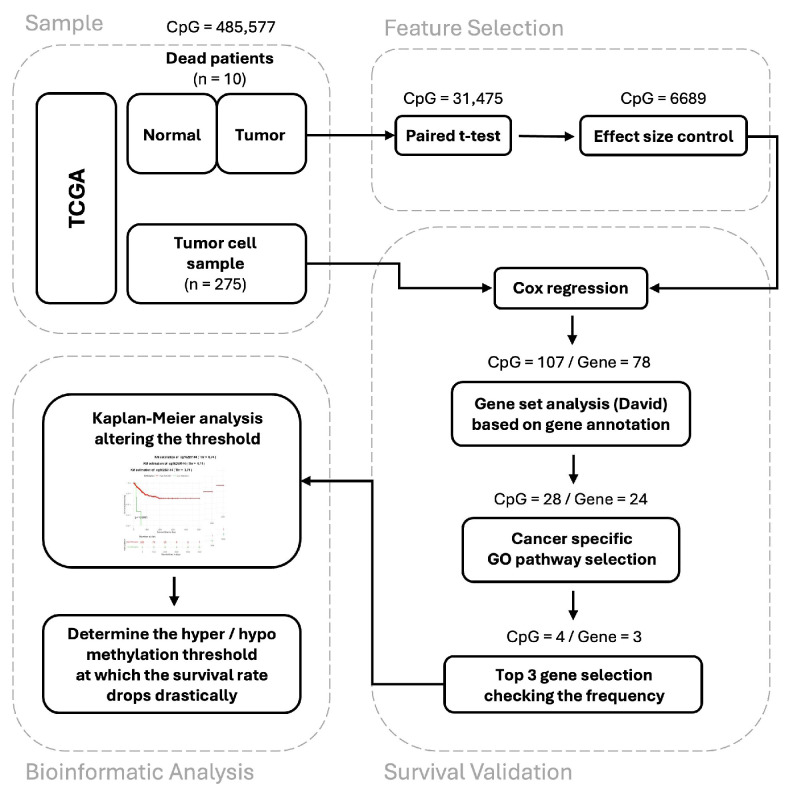
Overall workflow.

This study introduces a novel approach to prognostic prediction in bladder cancer (BLCA) using DNA methylation data. We developed a three-step strategy aimed at identifying biomarkers associated with poor prognosis (Figure 1). Initially, DNA methylation data were collected from the tissue samples of 285 BLCA patients. Among these, 10 deceased individuals had a matched adjacent normal tissue sample, while the remaining 275 patients contributed DNA methylation profiles from cancerous tissues.

In the first step of feature selection, we applied paired sample *t*-tests and controlled for effect size to mitigate the confounding factors that arose from genetic variability among individuals. Subsequently, in the second step of survival validation, we refined the candidate biomarkers by analyzing the significance of the test results, assessing the impact of CpG methylation levels on survival outcomes, and conducting gene set analyses.

In the final stage, we performed Kaplan–Meier survival analysis on a cohort of actual BLCA patients to establish a threshold for utilizing the selected biomarkers in prognostic predictions.

### 3.2. Identification of Candidate Biomarkers through Matched-Sample Analysis

We initiated this phase by employing matched-sample *t*-tests on DNA methylation data obtained from adjacent normal bladder tissue and bladder cancer tissue of the same individual. This approach aimed to pinpoint significant methylation changes specific to cancer cells while mitigating the influence of individual genetic differences. To address multiple testing problems, we applied a false discovery rate (FDR) correction set at a threshold of 0.01, identifying 31,475 CpG sites as statistically significant out of the 485,577 tested.

**Figure 2:**
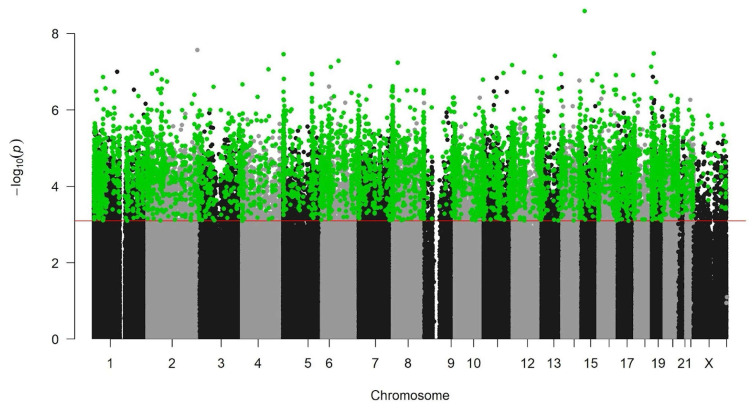
Visualization of the result of paired *t*-tests using the Manhattan plot. The x-axis denotes chromosome numbers, while the y-axis represents the −Log10-transformed *p*-values, indicating statistical significance. Each point corresponds to a CpG site, with green points and red lines indicating sites that passed the paired-sample *t*-test with an FDR threshold of 0.01, signifying non-overlapping methylation levels between normal and cancer cells. Black and gray points represent CpG sites where no significant difference was observed according to the aforementioned criteria.

Subsequently, we classified CpG sites into hypermethylated and hypomethylated groups relative to normal cells, focusing on CpG sites where methylation levels differed distinctly between normal and cancerous tissues. This stringent “effect size control” filtering resulted in the selection of 6689 CpG sites as candidate biomarkers (Figure 2).

### 3.3. Filtering with Survival Validation and Enriched Gene Set

Given the constraints of our initial sample size, it was imperative to validate both the statistical significance of our findings and the impact of CpG site methylation levels on survival outcomes. To achieve this, we performed Cox regression analysis using an independent dataset comprising preprocessed data from 275 BLCA patients. This dataset included survival status, survival time (median time to death: 352 days, median age: 72.63), the follow-up period (median follow-up: 527 days, median age: 70.26), and the methylation levels of the 6689 CpG sites identified in the previous step. From this analysis, we identified 107 CpG sites and 78 genes with a significance level below 0.01 (Supplementary Table S1), which were selected for further investigation.

Subsequently, we conducted a gene set analysis focusing primarily on the GOTERM_BP_DIRECT pathway. Among the biological processes (BPs) observed, we considered only those with significance levels of 0.05 or lower. Initially identifying 19 BPs, we excluded five processes that were ubiquitous across cell types, resulting in the final selection of 14 BPs (Table 2). Further analysis of gene frequencies within these 14 BPs highlighted that PRKCZ occurred seven times, PTK2 six times, and IL18 four times, indicating their significant roles across multiple biological processes. Consequently, these findings led us to select three genes and their associated four CpG sites as the final biomarker candidates for this study.

### 3.4. The Impact of Identified Markers with a Fixed Threshold on BLCA Survival

**Figure 3:**
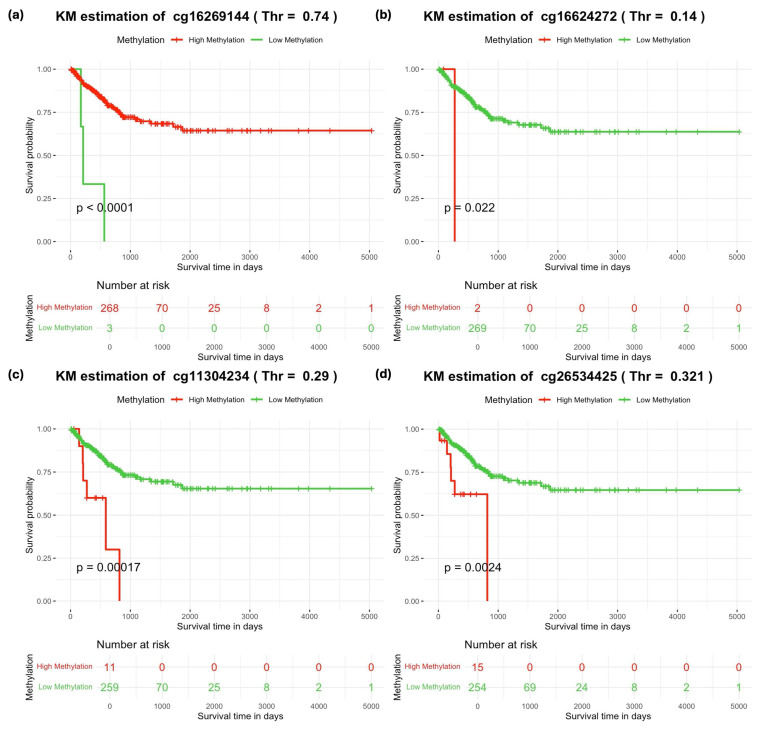
Kaplan–Meier plot illustrating the impact of hypomethylated and hypermethylated CpG biomarkers by selecting the optimal thresholds. In multiple plots, green and red lines represent hypomethylated and hypermethylated CpG status in cancer tissues, respectively. The x-axis denotes the survival time in days, while the y-axis indicates the survival rate. Subplots (**a**–**d**) demonstrate that specific CpG sites lead to fatal outcomes in bladder cancer patients when hypomethylated or hypermethylated, respectively.

We conducted Kaplan–Meier analysis to evaluate the influence of three genes on the survival of BLCA patients based on their methylation frequencies. The analysis revealed distinct survival patterns categorized by methylation status (death vs. survival) in binary groups (hyper- and hypomethylation). Each CpG site was assumed to have a baseline level of methylation, prompting us to adjust the threshold for binary classification and repeat the analysis. Interestingly, specific methylation values associated with certain genes emerged as critical thresholds that were linked to fatal outcomes in specific patient groups (Figure 3).

It is worth noting that four subjects out of the original 275 were excluded from the analysis due to missing CpG markers. For instance, for the cg16269144 marker associated with the PRKCZ gene, methylation levels below the threshold of 0.74 correlated with three patient deaths. Conversely, for cg16624272 linked to PTK2, methylation levels above the threshold of 0.14 led to two patient deaths. Lastly, for cg11304234 and cg26534425, associated with the IL18 gene, methylation levels exceeding the thresholds of 0.29 and 0.321, respectively, were linked with the deaths of 11 and 15 patients, respectively.

## 4. Discussion

Bladder cancer prognosis prediction remains a critical challenge in clinical practice, necessitating the discovery of novel biomarkers for accurate survival estimation. Moreover, research indicates a significant decline in health-related quality of life (HRQoL) among bladder cancer patients compared to cancer-free controls, emphasizing the ongoing need to address quality of life issues [17]. Our study proposes a strategy to uncover key prognostic biomarkers of bladder cancer by integrating DNA methylation analysis with matched samples from patients exhibiting extreme survival patterns (i.e., cancer and adjacent normal tissue samples).

Historically, studies faced limitations in addressing cancer heterogeneity by analyzing whole samples. Traditional systems, such as AJCC TNM staging, showed limited prognostic validation performance [18]. Although the European Association of Urology (EAU) NMIBC 2021 scoring model appears to be suboptimal in patients who undergo ReTUR and intravesical BCG therapy [19], these traditional systems are prone to interobserver variability and may not fully capture the molecular complexity of individual tumors. In contrast, our study stands out for its use of patient-matched samples, effectively addressing cancer heterogeneity. By analyzing DNA methylation data from adjacent normal bladder tissue and bladder cancer tissue from the same individual, we identified critical methylation changes specific to cancer cells while controlling for individual genetic differences.

Through DNA methylation analysis, we established distinct thresholds for methylation levels of CpG sites within pathways specifically associated with cancer. The analysis of the GOTERM_BP_DIRECT pathway provides insights into essential biological processes in cancer, including cell cycle regulation [20], apoptosis [21], DNA repair [22], metabolism [23], and immune response [24]. By identifying these key pathways, researchers can uncover potential biomarkers and therapeutic targets not only for bladder cancer but also for other malignancies. This approach highlights the interconnected nature of various biological processes and their collective impact on cancer progression and patient outcomes.

In our study, the analysis of genes PRKCZ, PTK2, and IL-18 underscores their significant roles in bladder cancer carcinogenesis, progression, and survival outcomes. PRKCZ, a member of the PKC family, acts as a tumor suppressor by inhibiting the NF-κB signaling pathway; its dysregulation contributes to uncontrolled cell growth and resistance to apoptosis, thereby adversely affecting patient survival when underexpressed [25]. PTK2 (FAK) plays a critical role in cell migration and invasion; its overexpression correlates with increased metastatic potential and poor survival outcomes, making it a promising therapeutic target [26,27,28]. IL-18, a pro-inflammatory cytokine, exhibits a dual role in cancer by enhancing anti-tumor immunity while promoting tumor growth and metastasis through a pro-inflammatory microenvironment, correlating with poor prognosis when elevated [29]. Understanding the functions of these genes provides crucial insights for developing targeted therapies and improving prognostic assessments in bladder cancer.

Kaplan–Meier analysis revealed distinct survival patterns associated with specific methylation beta values of certain genes when they reached defined thresholds, indicating adverse outcomes for patients. This highlights the significant impact of these genes under heterogeneous conditions in bladder cancer.

However, our study has limitations that warrant acknowledgment. The use of bladder tumors with matched normal tissue from the same subjects, while advantageous for controlling the genetic background, may limit the generalizability of our findings to the broader BLCA patient community. Additionally, the reliance on retrospective data and the inclusion of a small subset of extreme pattern data for feature selection introduced potential biases. These limitations underscore the necessity for further validation in larger, prospective cohorts to confirm the robustness and generalizability of our results.

Our study aimed to identify biomarkers that distinguish deceased patients from normal controls, pinpointing biomarkers that significantly impact survival. These findings elucidate critical molecular pathways associated with tumor progression, offering potential targets for personalized treatment strategies. By targeting hypermethylated genes with demethylating agents or specific inhibitors [30] and addressing hypomethylated genes through targeted suppression [31], we can mitigate tumor severity and enhance treatment efficacy. Implementing these findings could lead to individual, specific therapeutic interventions that optimize outcomes by precisely addressing each patient’s unique molecular profile [32]. By uncovering these biomarkers, we propose a novel approach to tailored prognosis and targeted therapy strategies for bladder cancer patients.

## Figures and Tables

**Table 1:** Characteristics of the participants used in the analysis.

Characteristics	*n* (%)	*n* (%)
No. Total	275	10
Sex		
Male	198 (72.0%)	6 (60.0%)
Female	66 (24.0%)	4 (40.0%)
NA	11 (4.0%)	0 (0.0%)
Age at Diagnosis		
30–40	2 (0.7%)	0 (0.0%)
41–50	14 (5.1%)	0 (0.0%)
51–60	55 (20.0%)	3 (30.0%)
61–70	79 (28.7%)	2 (20.0%)
71–80	80 (29.1%)	4 (40.0%)
≥81	34 (12.4%)	1 (10.0%)
NA	11 (4.0%)	0 (0.0%)
Race		
Asian	30 (10.9%)	0 (0.0%)
Black/African American	16 (5.8%)	1 (10.0%)
White	210 (76.4%)	8 (80.0%)
NA	19 (6.9%)	1 (10.0%)
Ethnicity		
Hispanic or Latino	8 (2.9%)	0 (0.0%)
Not Hispanic or Latino	238 (86.5%)	9 (90.0%)
NA	29 (10.6%)	1 (1.0%)
History of Other Malignancy		
Yes	70 (25.5%)	1 (10.0%)
No	194 (70.5%)	9 (90.0%)
NA	11 (4.0%)	0 (0.0%)
Vital Status		
Alive	191 (69.5%)	0 (0.0%)
Dead	73 (26.5%)	10 (100.0%)
NA	11 (4.0%)	0 (0.0%)
Histopathological Subtype		
Papillary	86 (31.3%)	1 (10.0%)
Non-Papillary	187 (68%)	9 (90.0%)
NA	2 (0.07%)	0 (0.0%)
Tumor Stage		
Stage I	1 (0.4%)	0 (0.0%)
Stage II	86 (31.3%)	2 (20.0%)
Stage III	93 (33.8%)	4 (40.0%)
Stage IV	94 (34.2%)	4 (40.0%)
NA	1 (0.4%)	0 (0.0%)
Tumor Grade		
T1	2 (0.7%)	0 (0.0%)
T2	16 (5.8%)	0 (0.0%)
T2a	22 (8.0%)	0 (0.0%)
T2b	38 (13.8%)	2 (20.0%)
T3	32 (11.6%)	4 (40.0%)
T3a	47 (17.1%)	1 (10.0%)
T3b	55 (20.0%)	1 (10.0%)
T4	4 (1.5%)	2 (20.0%)
T4a	29 (10.5%)	0 (0.0%)
T4b	4 (1.5%)	0 (0.0%)
NA	25 (9.1%)	0 (0.0%)
Smoking History *		
1	62 (22.5%)	3 (30.0%)
2	63 (22.9%)	3 (30.0%)
3	81 (29.5%)	1 (10.0%)
4	50 (18.2%)	0 (0.0%)
5	11 (4.0%)	0 (0.0%)
NA	8 (2.9%)	3 (30.0%)
Previous Therapy		
Yes	10 (3.6%)	0 (0.0%)
No	265 (96.4%)	10 (100.0%)

* Smoking History: lifelong non-smoker (less than 100 cigarettes smoked in lifetime) = 1, current smoker (includes daily smokers and non-daily smokers or occasional smokers) = 2, current reformed smoker for >15 years (greater than 15 years) = 3, current reformed smoker for ≤15 years (less than or equal to 15 years) = 4, and current reformed smoker, duration not specified = 5.

**Table 2:** Cancer-specific GOTERM_BP_DIRECT pathway selection.

Term	Count	*p*-Value *	Genes
GO:0010744~positive regulation of macrophage-derived foam cell differentiation	3	0.002+	PRKCH, IL18, NFKB1
GO:0031032~actomyosin structure organization	3	0.004−	EPB41L1, CDC42BPG, MYO18A
GO:0030010~establishment of cell polarity	3	0.006+	PRKCZ, PTK2, MARK2
GO:0030036~actin cytoskeleton organization	5	0.006+	CAPZB, PTK7, CDC42BPG, FHL3, ANTXR1
GO:0035556~intracellular signal transduction	6	0.013−	PRKCH, ASB13, TGFA, PRKCZ, MARK2, MAP4K4
GO:0030155~regulation of cell adhesion	3	0.017−	IL18, CYTH1, PTK2
GO:0060463~lung lobe morphogenesis	2	0.023−	LIF, GRHL2
GO:0045630~positive regulation of T-helper 2 cell differentiation	2	0.023−	IL18, PRKCZ
GO:0008284~positive regulation of cell proliferation	6	0.026+	LIF, GCNT2, TGFA, PRKCZ, PTK2, TRPM4
GO:0043066~negative regulation of apoptotic process	6	0.026−	MYO18A, PRKCZ, PTK2, NFKB1, LIMS2, MAP4K4
GO:0090179~planar cell polarity pathway involved in neural tube closure	2	0.036+	PTK7, GRHL3
GO:0007179~transforming growth factor beta receptor signaling pathway	3	0.044+	ZMIZ1, GCNT2, PTK2
GO:0016477~cell migration	4	0.046+	PTK7, MYO18A, PRKCZ, PTK2
GO:0032736~positive regulation of interleukin-13 production	2	0.049+	IL18, PRKCZ

* This table presents a list of significant gene sets based on *p*-values below 0.05, rounded to four decimal places.

## Data Availability

All reproducible codes and datasets are available upon request to the first authors or the corresponding author.

## Supplementary Materials

The following supporting information can be downloaded at https://www.mdpi.com/article/10.3390/medicina60071175/s1. Table S1: 107 Significant CpGs through cox regression analysis.
